# Is Korea Ready to Be a Key Player in the Medical Tourism Industry? An English Education Perspective

**Published:** 2020-02

**Authors:** Christian KIM, Song Yi LEE, Sam-Hun PARK

**Affiliations:** 1. Department of General Education, CHA University, Pocheon, South Korea; 2. Department of Airline Service, Dong Yang University, Yeongju, South Korea; 3. Asia Contents Institute, Konkuk University, Seoul, South Korea

**Keywords:** Medical tourism, English education, Nursing, Korea

## Abstract

**Background:**

This study attempted to identify the problem South Korea faces in its development of the medical tourism industry from the perspective of English education.

**Methods:**

To assess the preparedness and problems of future Korean nurses in dealing with foreign patients, a questionnaire was conducted in 2017 on 146 freshman and sophomore nursing major students at CHA University in South Korea.

**Results:**

Although the nursing major students were highly satisfied with the English instructors, they did not feel that the curriculum prepared them well to deal with foreign patients in the future. They also felt that the teaching methodology employed in the class should be changed to incorporate more medical content into the nursing English program.

**Conclusion:**

In order for South Korea to play a bigger role in the medical tourism industry, higher education institutions for medicine need to pay more attention to the English education of their students. More specifically, team-taught English for Specific Purposes (ESP) curricula should be established in order to meet the needs of future medical professionals.

## Introduction

The medical tourism industry is booming all over the world. It is estimated to generate annual revenues of US$60 billion, and is increasing at a rate of 20 percent every year (1). In order to obtain a piece of this increasing pie, many countries are making efforts to promote medical tourism.

Singapore is one of the countries that successfully led the medical tourism industry. It is a major tourist destination in Asia and serves as a geopolitical hub for Southeast Asia. The service industry in Singapore became internationalized due to the characteristics of its multi-ethnic population (2). In addition, the country actively recruited talented medical staff from abroad that can speak English as well as their other official languages. As a result, Singapore has the advantage of providing services to foreign patients without cultural and linguistic barriers.

India also emerged as a major medical tourism destination with approximately 150,000 medical tourists in 2005 (3). This market is estimated to grow at a rate of 30 percent per year (4). The strength of India’s medical tourism industry lies in its competitive pricing. In addition, its doctors and nurses are well-educated and communicate well with foreign patients since English is widely spoken in India.

South Korea’s medical technology is world-class, but it is not a key player in the Asian medical tourism market. One of the biggest reasons that Korea is not one of the main destinations for medical tourism is its language and communication problems. Unlike Singapore and India, Korea does not use English as a first language. This causes obvious problems between the Korean medical staff and foreign patients because the poor English proficiency can directly affect patient care. In one research, a medical tourism government official mentioned that most Korean doctors and administrative staff do not speak English and if some of them do, their English fluency is very low (5).

Another reason for the decline in the number of foreign patients coming to South Korea is the poor communication skills of its medical personnel. According to Rokni et al.’s research on identifying barriers that hinder the development of medical tourism in South Korea, communication skills was one of the main obstacles to increasing the number of foreign patients for medical tourism (6). Having communication skills as health practitioners means that they are able to communicate with cultural competence and provide appropriate services that are suitable for patients from diverse backgrounds.

Research has shown that primary caregiver communication is important for patient care. In Ferguson’s research, the patients’ well-being and health improved when the language barrier between the doctor and patient was reduced (7). Spanish-speaking Latino patients showed a correlation between doctor-patient language concordance and positive health outcomes. For example, one Latino patient with asthma who was cared for by a language-concordant physician went to the emergency room less, asked more questions and received more follow-up care than patients cared for by English-speaking physicians. Another Latino patient who was cared for by a language concordant caregiver also asked more questions and had more information recall.

Language also affects the relationship building between patient and physician. Rapport created through empathy and effective communication skills is needed to build a trusting relationship with the patient. These effective communication skills that help understand the patient’s symptoms and health beliefs improve patient satisfaction. There are studies that compare physician communication and rapport building skills with Latino patients and Caucasian patients (8, 9). The studies showed that Latinos who have limited English proficiency are less likely to build rapport with physicians, and are less likely to receive empathic responses from physicians.

Currently, the coordinator’s interpretation system causes communication problems between the patient and the primary caregiver and can further jeopardize the safety of the patient. In Ten’s research, findings revealed negative evaluations of medical tourists’ experiences in hospitals in Korea (10). One medical tourist believed that some coordinators were not qualified because they did not know the medical terminology and omitted important details in the translation. Another Russian client complained about a disastrous situation where the coordinator was unable to translate the simple word, *bone*, which led to unnecessary mistrust and suspicion.

Medical staff that treats medical tourism patients must be multilingual as well as understand different cultures. They also need to have knowledge in both medicine and medical tourism. Kim compared the attitudes of nursing students with other major college students toward multiculturalism (11). Surprisingly, non-nursing majors showed higher scores on multicultural attitudes than nursing majors, even though the nursing students had more experience dealing with foreigners. This means that there needs to be additional measures taken to promote positive multi-cultural attitudes of nursing students. One sure-fire way to better understand the culture of another country is to learn the language of that country.

The Korean Accreditation Board of Nursing Education defines the core competencies of nurses as: nursing service, training, counseling, research, leadership, and giving advice (12). Good communication skills are one of the essential skills needed to successfully use these competencies. Nursing is an action that involves interaction between the patient and the nurse. Since nursing services are delivered through communication, good communication skills is one the most important abilities for nurses (13). Also, open and accurate communication is important because nurses must communicate with patients, guardians, and staff in various departments (14).

Although communication skills are important for nurses, many nurses complain about communication conflicts between medical personnel and patients (15). Nurses who need to provide high quality patient care fail to provide comprehensive nursing care when there is unsuccessful communication between nurses and patients. When patients do not receive proper care, they are unsatisfied and furthermore, this will lead to negative experiences for nurses (16).

Communication skills refer to the ability to accurately convey one’s thoughts and feelings to others according to one’s intentions (17). If communication skills are insufficient, one’s intentions are wrongly transmitted and conflict with the other person occurs. Such interpersonal conflicts may also result in a lack of confidence in interpersonal relationships and deterioration in the quality of life (18). Due to poor communication skills, interpersonal conflict experiences may be a major stressor for nurses, affecting job performance, job satisfaction and turnover rates, and increasing the tension at work (19), thereby jeopardizing patient safety (20). Many nursing programs in universities in South Korea have already recognized the importance of communication skills and have set up courses within the curriculum to help nursing students improve their interpersonal skills and communication skills (21). This is a step in the right direction, however, the patients that nurses need to deal with in the medical tourism industry are not only Korean but foreigners as well. Therefore, nursing major students need to develop English communication skills and multicultural awareness required to deal with these patients.

Currently, there is a lack of research on the communication problem of medical manpower which is a weakness of the medical tourism industry in Korea. In most universities, the course evaluation is the only measuring stick for whether the medical English course was successful or not. To address this issue, the researchers of this present study attempted to identify the problems of English education of nursing college students who will be medical personnel in the future.

## Methods

We examined 146 freshman and sophomore nursing major students at CHA University, located in Pocheon, South Korea in 2017. Among the 75 freshmen, 17 were male and 58 were female with an age range of 18 to 22. There was a total of 71 sophomores consisting of 13 male and 58 female students with an age range of 19 to 25.

In the nursing program, the students are required to take a total of 16 hours of English during their first two years in university. This total is higher than the non-nursing majors, who only have to take a total of 12 hours of English classes. However, half of the required classes for the nursing students are test-preparation courses, which are taught by native Korean instructors in a lecture format with no English interaction. This means that the nursing students only take four, two-hour English courses with native English teachers before they graduate. The nursing majors take specialized English for Specific Purposes (ESP) classes titled, “Nursing English.” The native English teachers who teach these classes are very popular and the course evaluations given by the students are very high, therefore on the surface the nursing English program looks successful. However, the amount of time dedicated to learning English communication is extremely insufficient for the nursing students to handle foreign patients.

A questionnaire survey was conducted to measure attitudes and to identify potential problems in the English classes for freshman and sophomore nursing major students at a 4-year medical university. The data collected through the questionnaire was analyzed using SPSS 12.0 for Windows. The analyzing procedure is as follows: First, the data was classified into categories and all factors were analyzed through frequency analysis. A *t-*test was conducted to compare the means of the freshman and sophomore groups to see if there were any statistically significant differences. The results of the T-test showed that there were no significant differences between the freshman and sophomore students, thus the responses of the two groups were combined and analyzed as one large group.

The questionnaire used in this study was a closed questionnaire, based on the results of a pretest, the objective needs of Brindley (22), and the learning needs checklist of Hutchinson & Waters (23). The purpose of this survey was to identify the problems of nursing English classes and the needs of nursing major students for the development of medical tourism in Korea.

## Results

### Q1. Are you satisfied with your native English instructor?

The results for Q1 show that the students were satisfied with their English instructors, with 108 students (74%) responding either *strongly agree* (32 students) or *agree* (76 students). There were only 6 students (4.1%) who selected *disagree*. This result was expected since the native English instructors are the most popular professors on campus with extremely high course evaluation scores.

### Q2. Are you satisfied with the teaching methodology employed in your class?

For Q2, 95 students (65.1%) responded favorably with 27 students (18.5%) answering *strongly agree* and 68 students (46.6%), *agree*. Although only 4 students (9.1%) responded unfavorably, 47 students (32.2%) responded *neutral*, which may signal a need for improvements in the teaching methodology.

### Q3. Does this class help you communicate better in English?

For Q3, 15 students (10.3%) answered *strongly agree*, 48 (32.9%) answered *agree*, 72 students (49.3%) answered *neutral*, 10 students (6.8%) answered *disagree*, and 1 student (0.7%) answered *strongly disagree*. With only 63 students (43.2%) answering that the class was helpful for their English communication, we can see that the majority of the students did not find the class helpful.

### Q4. Does the class prepare you well for an encounter with foreign patients in the future?

There were 37 students (25.3%) who thought that the class prepared them well for a future encounter, with 6 students (4.1%) answering *strongly agree* and 31 students (21.2%) answering *agree*. There were 31 students who did not feel that the class prepared them well with 4 students (2.7%) *strongly disagreeing* and 27 students (18.5%) *disagreeing*. The most selected answer was *neutral* with 78 students (53.4%). The results from Q4 indicate that about three-quarters of the students do not think that the class gets them ready to deal with foreign patients in the future. This is a serious obstacle for the development of the medical tourism industry in Korea.

### Q5. What do you think is the most effective method to learn English in the classroom?

The most selected choice was *Team teaching with a nursing expert*, with 81 students (55.5%). This highlights a potential problem in the current nursing class, since the students may not feel that the native English teachers have enough medical expertise to teach medical content. In an ideal nursing English ESP class, one instructor would teach the medical content and the other instructor would teach the English language functions needed for a given medical situation. There were two second most selected responses, with 23 students (15.8%) choosing both *Medical situation roleplay* and *Utilizing multimedia*. The students’ response of *Medical situation roleplay* is related to the *Team teaching with a nursing expert* choice since the roleplays done in the class currently deal with non-medical, everyday situations. Also, the current nursing English classes rely heavily on reading materials, so the students’ request for more multimedia material is well called for.

### Q6. Is the number of required English class hours sufficient?

For Q6, 87 students (59.6%) responded that the current number of English class hours was sufficient. On the other hand, 23 students (15.8%) answered that the required hours were insufficient. 35 students (24%) did not take a side and chose *neutral* for this question. This result could have multiple implications. For the students that did not think that the required hours were sufficient, it is clear to see that they do not think that the current English courses prepared them well for English communication as nurses. For the students that answered that the hours were sufficient, they may mean that if the methodology were to be changed as in the results of Q5, the hours would be sufficient.

### Q7. Is there a need for additional nursing English classes in your junior and senior year?

The responses were split exactly in half with 72 students (49.3%) answering both *Yes* and *No*. It is difficult to make a recommendation for an increase in the number of English classes since the responses are not one-sided. However, for the students who answered *No*, it was not because they thought the number of English classes was sufficient, but because they had a heavy course load in their last two years and felt that they would not have enough time to take additional English classes. Nevertheless, at the present time, a change in the teaching methodology seems more urgent than an increase in the number of English classes.

## Discussion

Based on the results of the survey, we can conclude that although the nursing students are generally satisfied with the English instructors, the teaching methodology needs to be improved so that the needs of the freshman and sophomore students can be met. Also, the fact that *neutral* was the most selected response for the questions about whether the English classes helped their English communication and their ability to deal with foreign patients in the future, shows that a change in teaching methodology needs to be implemented.

In a research about stress factors of nurses, the language barrier was chosen as the biggest difficulty when dealing with foreign patients (24). This implies that nurses lack the communication skills to take care of foreign patients in the medical setting. Therefore, any higher education institution that fosters medical experts needs to plan courses that deal with actual communication in real situations. In other words, after assessing the needs of the future medical personnel, a systematic ESP program needs to be established.

English programs in Korean universities began teaching general English conversation in the form of English for General Purposes (EGP) classes. After that, universities started to teach major content courses in English, giving rise to the notion of content-based education in English (25). This led to the birth of English for ESP classes that focus on the practical and professional needs of the students as well as the English language (26). Since the jargon and language needs vary from field to field, an ESP education focusing on the communication needs of a specific field is called for (27).

The most desired teaching methodology was *Team teaching with a nursing expert*. This is supported by the findings of a research on students in the administrative assistant department, which stated that optimal benefit from ESP classes can be achieved when a language expert team teaches with an expert with actual experience in the field of study (28). This will alleviate the pressure on the English instructors to master the contents of the nursing field, which may take an indefinite amount of time.

Finally, since the number of students who want more English classes are the same as the number of students who do not, it is premature to recommend any changes in the total number of class hours. For the time being, a change in how the classes are taught seems to be more urgent.

## Conclusion

Since many nursing programs in South Korean universities have similar English education curricula to the university in the present research, it can be concluded that universities are not preparing future medical professionals to play a major role in the medical tourism industry with regard to English proficiency and communication skills. To solve this problem, nursing programs need to incorporate ESP nursing curricula that can meet the needs of their students. Since English instructors are not likely to have in-depth medical expertise, classes need to be co-taught with an experienced nursing professional who can provide the medical content. That way, the students can receive both the nursing and English content. The implications of this study are of great significance in providing guidance on what efforts need to be made by higher education institutions that produce medical professionals so that they can contribute to the revitalization of the medical tourism industry by effectively communicating and dealing with foreign patients.

## Ethical considerations

Ethical issues (including plagiarism, informed consent, misconduct, data fabrication and/or falsification, double publication and/or submission, and redundancy) have been completely observed by authors.
